# Second-Order Regression-Based MR Image Upsampling

**DOI:** 10.1155/2017/6462832

**Published:** 2017-03-30

**Authors:** Jing Hu, Xi Wu, Jiliu Zhou

**Affiliations:** Department of Computer Science, Chengdu University of Information Technology, Chengdu 610225, China

## Abstract

The spatial resolution of magnetic resonance imaging (MRI) is often limited due to several reasons, including a short data acquisition time. Several advanced interpolation-based image upsampling algorithms have been developed to increase the resolution of MR images. These methods estimate the voxel intensity in a high-resolution (HR) image by a weighted combination of voxels in the original low-resolution (LR) MR image. As these methods fall into the zero-order point estimation framework, they only include a local constant approximation of the image voxel and hence cannot fully represent the underlying image structure(s). To this end, we extend the existing zero-order point estimation to higher orders of regression, allowing us to approximate a mapping function between local LR-HR image patches by a polynomial function. Extensive experiments on open-access MR image datasets and actual clinical MR images demonstrate that our algorithm can maintain sharp edges and preserve fine details, while the current state-of-the-art algorithms remain prone to some visual artifacts such as blurring and staircasing artifacts.

## 1. Background

Magnetic resonance (MR) imaging is widely used to assess brain diseases, spinal disorders, cardiac function, and musculoskeletal injuries. Compared with computed tomography, MR imaging requires a longer acquisition time [1]. Hence, in order to minimize involuntary patient motion in MR imaging, the scan time is often shortened, thereby obtaining MR images with fewer slices and larger spacing [2]. In other words, MR images are usually highly anisotropic (e.g., 1 × 1 × 6 mm^3^), with a lower resolution in the slice-selection direction than in the in-plane direction [3]. However, in many medical applications, an isotropic MR image is required [4]. In addition, a higher resolution of MR image is also essential for more detailed understanding of human anatomy, facilitating early detection of abnormalities, and improving clinical assessment accuracy [4].

A possible approach to increase MR image resolution is an interpolation-based image upsampling [2]. Traditional interpolation methods adopted in natural image processing field such as spline interpolation can be directly employed. But these methods use fixed interpolation coefficients and only select spatially nearby sampling voxels, thereby producing images with blurred edges and staircasing artifacts. To reduce these unwanted artifacts, some sophisticated interpolation methods [2, 5–11] have been recently proposed in biomedical image processing. Largely, these advanced interpolation methods are derived from a nonlocal redundancy concept [12]. That is, they specifically select sampled voxels or adapt interpolation coefficients. For instance, Manjón et al. [5] proposed determining the interpolation coefficients through the similarity of intensities between two 3D image patches around the unknown voxel and sampled voxels. Observing that image structures in a slice-selection direction of a 3D MR image also exist in an in-plane direction, while the resolution of the latter is usually higher than the former's, Plenge et al. [6] proposed reconstructing a high-resolution (HR) version in a slice-selection direction by leveraging cross-scale self-similarity and cross-scale resolution discrepancy from patches in in-plane direction. Furthermore, Qu et al. [7] extended Plenge's work by using the sparsity of similar image patches. Besides the methods proposed in [5–7] which use only the input low-resolution (LR) image, several other algorithms have also been advocated to leverage HR images of the same subjects in other imaging modalities, with the aim of reconstructing more high-frequency image details. For example, a combined interpolation weight proposed in [8] was calculated from both an example HR image and an LR input image. Further, Manjón et al. [9, 10] developed a correlation map which shows the inconsistencies between HR image and LR input so as to adjust the relative importance of these two images in the combinational weight computation. On noticing that the voxel similarity comparison via Euclidean distance of intensity was not rotationally invariant, Jafari-Khouzani et al. [2, 11] proposed to concatenate several image features computed from each voxel, such as gradients and Gaussian-blurred intensity values, into a vector and measure the voxel similarity from this feature vector therein.

Intuitively, whether using a different imaging modality or not, the above-mentioned advanced interpolation algorithms all focus on the refinement of interpolation weights. Nevertheless, using a weighted averaging scheme, these algorithms essentially can be described as a zero-order regression problem [13]. Unfortunately, the zero-order regression prototype risks blur the laminar shape of brain structure, and therefore high-order image details cannot be well retrieved [2, 13]. To the best of our knowledge, few efforts have been made in MR image processing to promote an interpolation algorithm that preserves higher-order details.

To address the fine-detail limitation in current interpolation algorithms, we propose a regression-inspired interpolation method using second-order polynomials. Under a kernel regression framework, we attempted to transform an LR image space into the expected HR image space by establishing a set of high-order prototypes so as to locally approximate the ground truth image space. Moreover, to further strengthen local consistency in the proposed method, a patch-based image reconstruction scheme is utilized rather than voxel-by-voxel scheme as other interpolation methods do. With these two salient features, the proposed method successfully enhances high-frequency details in final results.

In the rest of this paper, the proposed method will be specified in Section 2. Extensive experimental results and discussions are demonstrated in Section 3. Finally, a conclusion is described in Section 4.

## 2. Methods

In this section, we firstly describe a MR image acquisition model and mathematical notations therein and then present the specific implementation of our regression-based image upsampling algorithm.

### 2.1. The Acquisition Model and Notations

In the context of MR imaging, the most accepted image acquisition model assumes that an acquired LR image is generated from an HR counterpart through a sequence of degradation operations, such as motion blur, field inhomogeneity, and noise, and can be represented as [1]

(1) $$\begin{array}{c} Y=DHZ+\eta , \end{array}$$

where **Y** denotes an observed LR image, **D** is a downsampling operator, **H** is a blurring operator, **Z** is an HR image, and **η** is acquisition noise, which is often regarded as Rician-distributed in MR imaging. To infer **Z** from **Y**, image upsampling is basically an inverse process of MR imaging. Moreover, owing to the fact that the specific form of the blurring operation is unknown, this blurring operation could be regarded as an unspecified mapping function. In light of this, the HR image could then be formulated as

(2) $$\begin{array}{c} Z=f\left(\;D^{T}\tilde{Y}\;\right), \end{array}$$

where **D**^*T*^ is a simple interpolation operator (i.e., bicubic interpolation) which generates the initial estimation of the upsampled image and $\tilde{Y}$ is the denoised version of the LR image **Y**; the mapping function *f* denotes the inversed blurring operation, representing the relations from an LR space to the target HR space.

Equation (2) translates the image upsampling problem into a regression problem, with the particular form of regression function *f*(*▪*) remaining unspecified. To estimate *f*(*▪*), we propose the development of a generic local expansion of *f*(*▪*) about one image patch in $D^{T}\tilde{Y}$ image. This particular method will be discussed in Section 2.2.

**Figure 1:**
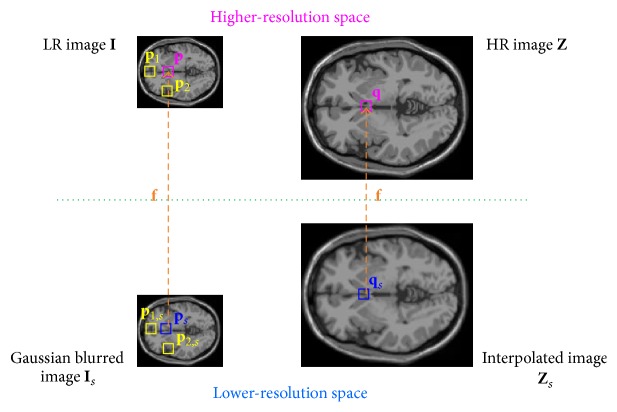
The patch relations of the proposed method.

As shown in Figure 1, the goal of the proposed method is to construct an HR version **Z** from a given LR image **I** (which is in fact image $\tilde{Y}$ in (2)). **I**_*s*_ is a smoothed version of **I** (where *s* represents smoothing) and **Z**_*s*_ is an upscaled version of **I**. The bolded lowercase **p** and **q** denote the column vectors of two *s* × *s* image patches which are extracted from **I** and **Z**, respectively; **p**_*s*_ and **q**_*s*_ are the column vectors of two *s* × *s* image patches taken from **I**_*s*_ and **Z**_*s*_, respectively. Among all patches in image **I**_*s*_, **p**_*s*_ is the most similar one to **q**_*s*_; patches **p**(**q**) and **p**_*s*_(**q**_*s*_) have the same coordinates for the center pixel. In the proposed method, {**p**_*s*_, **p**} constitutes LR-HR training patch pairs and {**q**_*s*_, **q**} constitutes LR-HR testing patch pairs.

### 2.2. Regression-Based Image Upsampling Method

#### 2.2.1. HR Patch Representation Based on a Regression Model

Learning the regression function (or mapping function) in (2) is extremely difficult because of its ill-posed nature. To constrain its solution space, proper regularization is usually required. Multiscale image self-similarity property has been used as effective regularization in several ill-posed problems of image processing, such as image denoising [12] and image superresolution [14]. More specifically, the multiscale image self-similarity property refers to the recurrence of image patches, and, generally, it includes two parts: nonlocal self-similarity within one scale [12] and that across scales. Recently, the validity of the nonlocal self-similarity in one scale has been successfully verified for MR images, including T1W images [2, 5] and DWI images [10]. Although self-similarity across scales has not been as widely used in MRI as self-similarity in one scale, its existence is apparent, since the primary structure of interest is assumed not to be lost when an image is downsampled, as confirmed on natural images in [15]. In this sense, multiscale self-similarity property can also be extended into MR images with abundant textures, such as images of brain, liver, and heart.

In this paper, the multiscale self-similarity property is leveraged in two ways. Firstly, since an image is likely to have repeated patterns, the mapping function *f* in (2) is estimated patch-wisely in the proposed method rather than being estimated from the entire image. With this regarding, *f* associates each LR-HR patch pair {**q**, **q**_*s*_} as **q** = *f*(**q**_*s*_). Secondly, the fact that singular structures are scale invariant implies that each patch **q**_*s*_ in image **Z**_*s*_ could find its similar patch **p**_*s*_ in image **I**_*s*_. In other words, the mapping function of patch **q**_*s*_ to its high-resolution counterpart **q** can be regarded as the same mapping function of patch **p**_*s*_ to patch **p**. In light of this, a set of {**p**_*s*_, **p**} can be served as a sort of adaptive patch regularization to infer *f* and consequently the high-resolution version of **Z**_*s*_.

More specifically, to estimate the function *f* for patch **q**_*s*_, a local expansion of *f* on patch **q**_*s*_ could be developed by utilizing an *N*th-order Taylor series, if patches **q**_*s*_ and **p**_*s*_ are similar:

(3) qfqs=fps+qs−ps=fps+f′ps∘qs−ps+12f′′ps∘qs−ps∘qs−ps+⋯≈p+f′ps∘qs−ps+12f′′ps∘qs−ps∘qs−ps,

where ∘ denotes the element-wise product of two matrices; *f*′(·) and *f*′′(·) denote the first and second derivatives of the mapping function *f*. It is easy to tell from (3) that, by addressing the image upsampling problem as one of kernel regression, we are able to generalize the intensity of HR patch **q** to arbitrary orders, which gives us greater flexibility in modeling the underlying image data. On the other hand, (3) also reveals that, in order to reconstruct the HR patch **q**, multiorders of the mapping function derivatives should be estimated first. Several authors [16, 17] have argued in their work that, for image reconstruction, a second-order derivative estimation is able to adequately balance detail preservation and computation time. Therefore, we propose a second-order derivation of the mapping function in this method.

#### 2.2.2. Estimation of Mapping Function's Derivative Using Local Self-Similarity

Image self-similarity property also reveals that, inside image **I**_*s*_, patches with a similar layout to the patch **p**_*s*_ can also be explored (see yellow boxes **p**_1,*s*_ and **p**_2,*s*_ in Figure 1). Therefore, the mapping function of the patch **q**_*s*_ to its high-resolution counterpart **q** can also be regarded as the same mapping function of patch **p**_1,*s*_ to patch **p**_1_, where **p**_1_ is high-resolution counterpart of **p**_1,*s*_ in image **I**. Like (3), the mapping function on patch **p**_1,*s*_ could also be locally expanded as

(4) p1fp1,s=fps+p1,s−ps=fps+f′ps∘p1,s−ps+12f′′ps∘p1,s−ps∘p1,s−ps+⋯≈p+f′ps∘p1,s−ps+12f′′ps∘p1,s−ps∘p1,s−ps.

From (3) and (4), we can see that *f*′(·) and *f*′′(·) are both derived from local signal representations. Thus, it is reasonable to estimate these two parameters using all the “neighboring” patches of **p**_*s*_ in terms of patch content. In light of this, by incorporating the *J*-most similar patches {**p**_*i*,*s*_}_*i*=1_^*J*^ and their paired HR patches {**p**_*i*_}_*i*=1_^*J*^, we can learn the function *f* in a weighted-least-square formulation:

(5) minf′ps,f′′ps ∑i=1Jpi−p−f′ps∘pi,s−ps−12f′′ps∘pi,s−ps∘pi,s−ps22wpi,s−ps,

where *w*(**p**_*i*,*s*_ − **p**_*s*_) measures the similarity between patches **p**_*i*,*s*_ and **p**_*s*_. To effectively characterize the contained brain structures, we represent each patch by its region covariance descriptor [2]:

(6) $$\begin{array}{c} Cp_{s}=\frac{1}{P}\overset{P}{\underset{i=1}{\sum }}\left(\;f_{i}-\mu \;\right){\left(\;f_{i}-\mu \;\right)}^{T}, \end{array}$$

where *P* denotes a total number of pixels in patch **p**_*s*_, **f**_*i*_ is a feature point of each voxel inside **p**_*s*_, and ***μ*** is the mean value of overall feature points. Regarding the feature point, simple visual features such as intensity and spatial information are adopted in our method, and **f**_*i*_ is hence calculated as

(7) $$\begin{array}{c} f_{i}={\left[\;{Ι}_{i},\frac{\partial I_{i}}{\partial x},\frac{\partial I_{i}}{\partial y}\;\right]}^{T}, \end{array}$$

where **I**_*i*_ is the intensity value of voxel* i* and ∂/∂*x* and ∂/∂*y*, respectively, represent the gradients along horizontal and vertical directions. Note that covariance matrices do not lie in Euclidean space; thus a metric proposed in [18] is utilized to compute the similarity between two covariance matrices:

(8) $$\begin{array}{c} w\left(\;p_{i,s}-p_{s}\;\right)=\exp\left(\;\frac{-\sum_{j=1}^{N}ln^{2}\lambda_{j}\left(Cp_{i,s},Cp_{s}\right)}{2}\;\right), \end{array}$$

where {*λ*_*j*_(**Cp**_*i*,*s*_, **Cp**_*s*_)}, *j* = 1,…, *N*, are the generalized eigenvalues of **Cp**_*i*,*s*_ and **Cp**_*s*_, respectively, and *N* equals the number of columns in patch **p**_*s*_.

**Figure 2:**
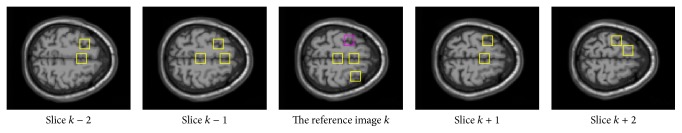
Example of nonlocal self-similarity property inside one single image and its nearby slices. The pink square region represents the reference patch and the green square regions are the similar patch found in the same image and the nearby images.

Returning to the optimization problem in (5), it is obvious that this formula should be overdetermined to obtain reliable and valid solutions. More specifically, the number of equations should exceed the number of unknown derivative coefficients which is related to the size of **p**_*s*_. For instance, for a 5 × 5 patch **p**_*s*_, the total number of coefficients in *f*′(·) and *f*′′(·) is 50, which implies that more than 50 similar patches of **p**_*s*_ should be found to obtain a solid estimation. Obviously, finding such a large number of similar patches inside one single image is impossible. But, fortunately, due to the nature of MR images, similar anatomical features still occur in nearby MR slices (see Figure 2). In this sense, similar patches **p**_*i*,*s*_ in the adjacent image slice are also exploited to *f*′(·) and *f*′′(·) estimation.

 By denoting

(9) y=p1,s−psp2,s−ps⋮pJ,s−ps,W=diag⁡wp1,s−ps×1diag⁡wp2,s−ps×1⋮diag⁡wpJ,s−ps×1,X=diag⁡p1,s−psdiag⁡p1,s−ps∘p1,s−psdiag⁡p2,s−psdiag⁡p2,s−ps∘p2,s−ps⋮⋮diag⁡pJ,s−psdiag⁡pJ,s−ps∘pJ,s−ps,

with 1 denoting a column vector with all elements equal to one and diag⁡(*▪*) defining a diagonal matrix, (5) is then expressed in the following matrix form:

(10) $$\begin{array}{c} \underset{b}{min}{\left\|\;y-Xb\;\right\|}_{W}^{2}, \end{array}$$

where

(11) $$\begin{array}{c} b={\left[\;f^{\prime }\left(\;p_{s}\;\right),f^{\prime \prime }\left(\;p_{s}\;\right)\;\right]}^{T}. \end{array}$$

Using weighted-square estimation, a closed-form solution of (10) is obtained: $\hat{b}={\left(X^{T}WX\right)}^{-1}X^{T}Wy$.

Once *f*′(·) and *f*′′(·) are estimated, they are added back to (3) to obtain the HR patch **q**. In the same way, the whole HR image is reconstructed patch-wisely, where estimators on overlapped regions are simply averaged. Next, a mean-correction step as advocated in [2, 5, 11] is applied to ensure the consistency between a reconstructed HR image and the original LR input.

 An overview is presented in Algorithm 1.

## 3. Experiments and Discussions

The proposed framework was evaluated using some state-of-the-art algorithms in both synthetic and clinical MRI datasets. Nearest neighbor (NN) interpolation, nonlocal means (NLM) based upsampling [5], and Gaussian process regression (GPR) based upsampling [19] were employed for comparison. The implementation of NLM and GPR was made available by their authors, and the free parameters inside these two approaches were selected based on authors' suggestion. Note that the NLM method belongs to a zeroth-order estimation, and an iterative process is employed to refine interpolation weights (also zero-order-based). The proposed method, however, belongs to second-order estimation and does not need iterative procedures for desirable effects. Therefore, to make a fair comparison between the proposed method and NLM method, the components other than derivative order should be the same. In this way, improved performance of our method can be solely attributed to the advocated second-order regression scheme. Hence, the numbers of iterations in both NLM and our method were chosen to be the same. Additionally, we reported and compared the output from the first iteration of NLM in the following experiments, with the aim of avoiding other complex factors introduced from more iterations such as cumulative errors.

Three open-access datasets, namely, BrainWeb [20], National Alliance for Medical Image Computing (NAMIC, real images, http://hdl.handle.net/1926/1687), and Human Connectome Project (HCP, real images, https://db.humanconnectome.org/), were selected for comparison. In addition, a real clinical MRI dataset was also used for evaluating the adaptability of the proposed framework in a realistic scenario, where subjects gave informed consent to participate, and recordings were used according to the study purpose.

In this paper, LR volumes were generated in two steps: blurring and downsampling. In MRI, blurring along the slice direction is related to the radio frequency pulse and gradient waveform, and it generally accepted the fact that such blur kernel could be well approximated by a Gaussian function in three dimensions [1]. Therefore, in this paper, the blurred image was generated by convolving the HR image with a 3D Gaussian kernel with a standard deviation of 0.8 (in voxel space) along dimensions. Next, the blurred images were downsampled to lower voxel resolutions, such as 2 × 2 × 2 mm^3^ and 1 × 1 × 6 mm^3^.

Three quality measures were used to evaluate the performance of different upsampling methods. The first was Peak Signal to Noise Ratio (PSNR), which is a noise level measurement commonly used in image processing. The second metric was Structural Similarity (SSIM) [21], which is a measurement assessing the quality perception of the human visual system. The third metric is mutual information (MI), which measures dependence between two images. A high PSNR score indicates that a resultant image contains little distortion and few noises; a SSIM value near 1 and a high MI value imply that the reconstructed image is close to the ground truth.

### 3.1. Parameter Settings

The proposed high-order regression-based method has four free parameters. These are the radius (*v*) of the search region, the radius (*r*) of the 2D patch used to learn the mapping function between different resolutions, the number (*p*) of the neighboring slices used to find the similar 2D patches **p**_*s*_, and the number (*J*) of the most similar 2D patches from each slice used to learn the mapping function's second-order derivative. Parameters* v* and* r* were set to 11 and 2, respectively, and 5 image slices were used to find the similar patches. For parameter *J*, *p* × *J* should be higher than (2*r* + 1)×(2*r* + 1) × 2 to ensure a valid second-order derivative prediction. Hence, in this paper, *J* was set to 11. In addition, the interpolated image **Z**_*s*_ was generated from the input LR image by upsampling with bicubic interpolation. Regarding the blurred image **I**_*s*_, it was generated by downsampling and upsampling the denoised LR image $\tilde{Y}$ with bilinear interpolation.

### 3.2. Phantom Data Evaluation

#### 3.2.1. BrainWeb Dataset

For this experiment, five isotropic T1 volumes (voxel resolution 1 mm^3^, 180 × 216 × 180 voxels), corrupted by Rician noise at different noise levels (0%, 3%, 5%, 7%, and 9% of the maximum intensity), were initially downloaded from BrainWeb phantom. These five T1 volumes were used as the HR volumes in the following experiments. Each of these five volumes was blurred and downsampled to 2 × 2 × 2 mm^3^ or 3 × 3 × 3 mm^3^ resolutions. To evaluate the effectiveness of the proposed method, simulated LR images were upsampled to 1 mm isotropic resolution using the proposed and compared upsampling methods.

**Figure 3:**
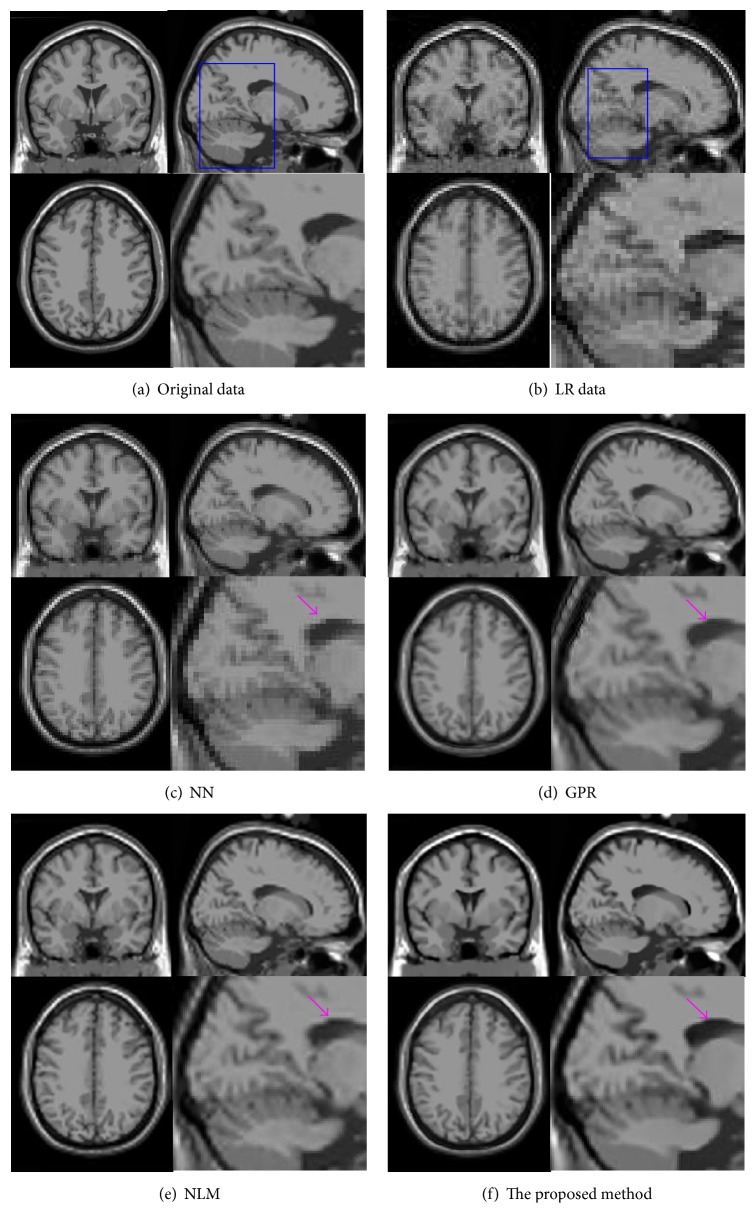
Upsampling results (1 × 1 × 1 mm^3^) for simulated data with 3 × 3 × 3 mm^3^ on BrainWeb.

Figure 3 demonstrates the upsampling results using the LR images generated from noise-free T1 volumes, where a typical slice is shown at coronal, sagittal, and axial views. Note that the sizes of these resultant images are reduced due to space limitations. For this reason, we also provided some close-up zones in the sagittal view for a better visual comparison. As can be observed from Figures 3(c)–3(f), a simple NN interpolation method introduces ringing and jaggy artifacts, while GPR, NLM, and the proposed method all effectively produce visually superior performance. This is because the latter three methods have a greater flexibility in modeling the underlying image data. However, using a zeroth-order estimation, GPR method and NLM method tend to produce ghost-like edges (see Figures 3(d) and 3(e)), whereas no obvious artifacts were observed from the proposed method. This phenomenon implies that employing the second-order regression strategy is a promising option for generating high-frequency details.

**Figure 4:**
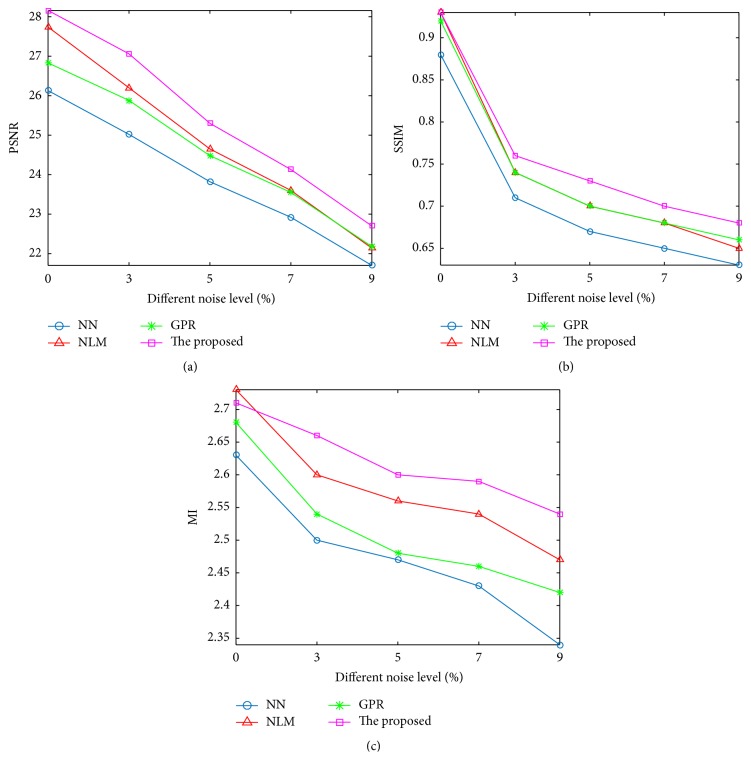
Upsampling results on simulated data (2 × 2 × 2 mm^3^) with different noise levels. (a) PSNR results. (b) SSIM results. (c) MI results.

Further, experiments on noisy LR images were also conducted to characterize how noise affects the image upsampling algorithms. Since the Gaussian regression in GPR can somewhat reduce image noise, this method can be directly performed on noisy data. Regarding the other upsampling methods like NLM and ours, noisy data was first filtered using APW-NLM method [22] that employs a strategy of adaptive bandwidth and patch size. Subsequently, all SR methods except GPR method were applied to these filtered data. The upsampled HR images were then compared with the original noise-free volume.  Figure 4 presents PSNR, SSIM, and MI measurements for all methods on LR images with a slice thickness of 2 mm while changing noise level to 0%, 3%, 5%, 7%, and 9%. It can be clearly observed from Figure 4 that, at the vast majority of noise levels, the proposed method outperforms the other algorithms under comparisons. In addition, we also observe that the PSNR and SSIM performance gaps between the proposed method and the compared algorithms increase further when noise level is increased. Moreover, it is interesting to note that when LR input image is noise-free, NLM method provides a PSNR gain of 1 dB over GPR method, which demonstrates the effectiveness of NLM method. However, when LR input images become noisy, for example, with a noise level at 9%, NLM method provides an inferior quantitative performance in terms of PSNR and SSIM. In other words, the performance gap between NLM and GPR decreases with an increasing noise level, which implies the potential of simultaneous interpolation and denoising in case of noisy images.

#### 3.2.2. NAMIC Dataset

**Figure 5:**
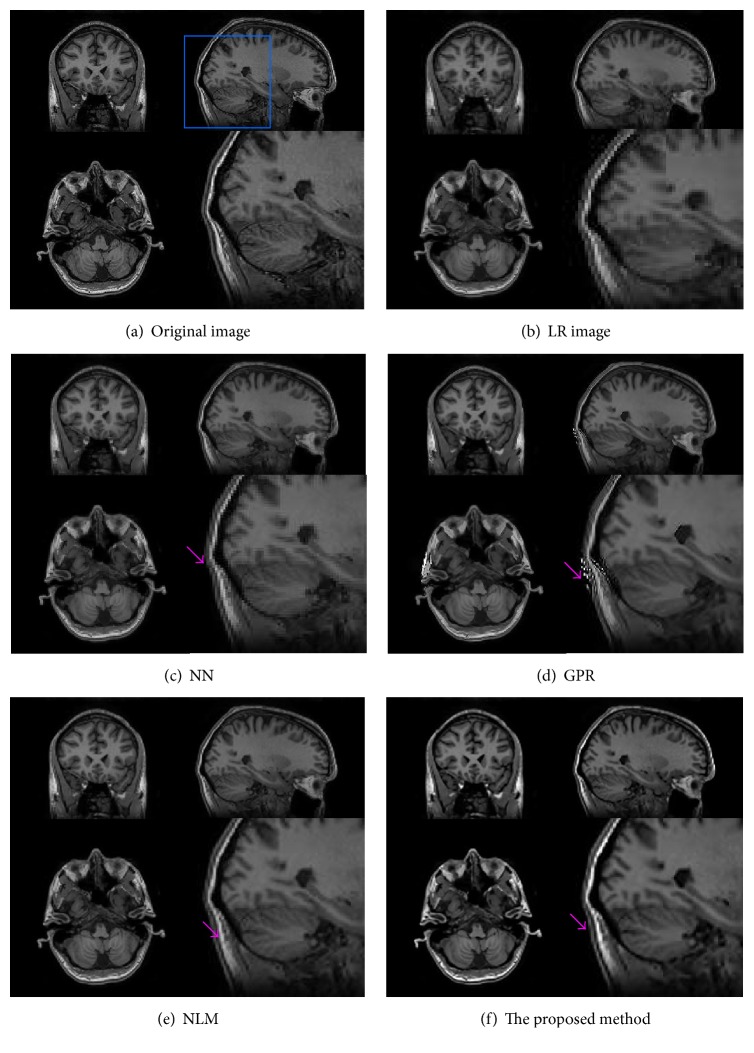
Upsampling results for simulated data on NAMIC. Results of LR data with 1 × 1 × 2 mm^3^ resolution upsampled to 1 × 1 × 1 mm^3^.

The NAMIC dataset included 10 schizophrenic patients and 10 normal controls from various imaging modalities. In this experiment, we randomly selected one example of T1W volumes from these twenty T1W sources with 1 mm isotropic resolution (matrix size: 256 × 256 × 176) as the initial HR images. LR images were generated by 3D Gaussian blurring and then downsampled to 1 × 1 × 2 mm^3^, 1 × 1 × 3 mm^3^, 1 × 1 × 5 mm^3^, and 1 × 1 × 6 mm^3^. The downsampled T1W images were then upsampled using the proposed method and the compared methods.  Table 1 illustrates the corresponding PSNR, SSIM, and MI values of these upsampling results, and Figure 5 presents an example of the upsampling result of an axial view for 1 × 1 × 2 mm^3^ upsampling, together with a magnified zone for better visualization.

From Table 1, we see that the proposed method gives superior performance on all the anisotropic low resolutions. Regarding Figure 5, the NN method still produces jagged edges. Although the GPR method provides sharper edges than the NLM method and the proposed method, it tends to cause overshoot artifacts along edges. In contrast, both NLM method and the proposed method demonstrate robust performance. Nevertheless, as clearly illustrated in the magnified region, the proposed method produces more vivid details than the NLM method; that is, the laminar structures are perceptually salient. This advantage can be mainly attributed to the high-order regression technique employed in the proposed method.

#### 3.2.3. HCP Dataset

The HCP dataset contains an enormous amount of multimodal data (such as fMRI and structural MRI), including T1W data types that were acquired on a Siemens 3 T scanner [TE = 2.14 ms, TR = 2400 ms, and TI = 1000 ms] and processed by a structural preprocessing pipeline. In our experiment, five subjects were randomly selected from this dataset, and each one had a T1W volume (matrix size: 260 × 311 × 260) with 1 mm isotropic voxel size. To generate LR images, the T1W volume was blurred and downsampled to a resolution of 2 × 2 × 2 mm^3^.

**Figure 6:**
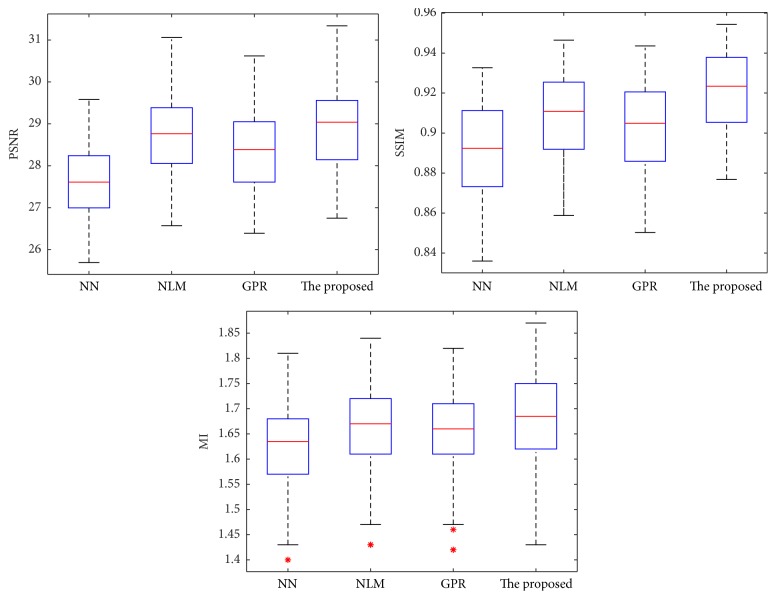
Boxplot of PSNR, SSIM, and MI results for reconstructing T1W LR images from the HCP database using different methods. *∗* denotes the outlier.

PSNR, SSIM, and MI values for these five test groups using different upsampling methods were shown as boxplots in Figure 6. The bottom and top of the boxes are the 25th and 75th percentiles, the bold band near the middle of the box is the median, and the whiskers extending from each box show the whole of the rest of the data. As shown by boxplots, the proposed method significantly outperforms all comparison methods.

### 3.3. Real Data Evaluation

**Figure 7:**
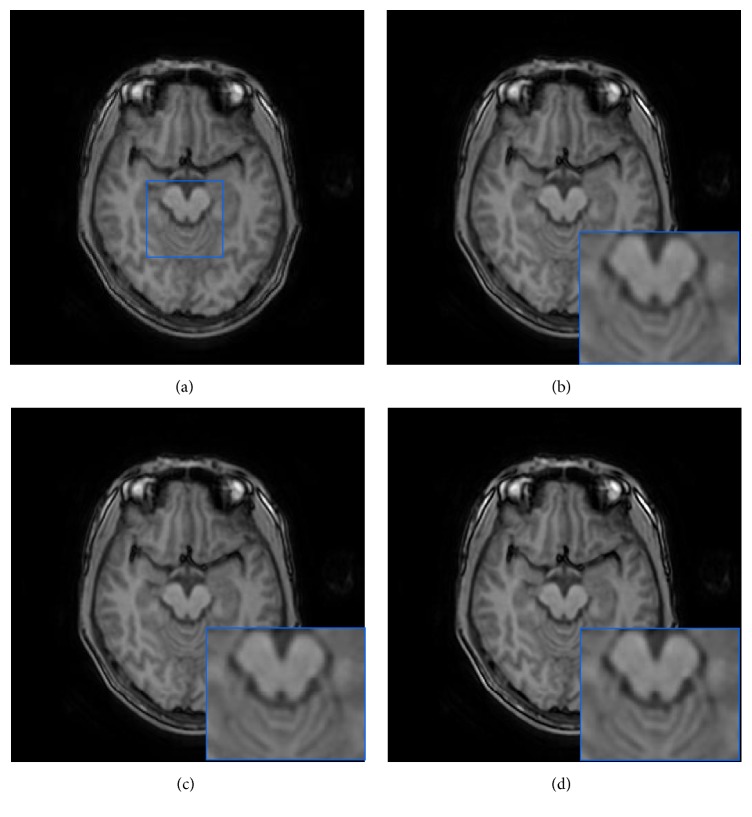
Comparison of upsampling results on real clinical data. (a) LR image slice. (b)–(d) are image upsampling results from GPR method, NLM method, and the proposed method, respectively.

In addition, we further applied our method directly to some real clinical MRI images which were obtained by a GE MR750 scanner. In this case, an LR T1W volume (256 × 256 × 78 voxels) with a voxel resolution of 2 × 2 × 2 mm^3^ was obtained. In our experiment, these LR T1W data were upsampled to 1 mm^3^ using the NLM method, the GPR method, and the proposed method. The reconstructed results were visually compared in Figure 7. It can be clearly seen that the upsampled volume using the proposed method is significantly less blurry and contains sharper edges; that is, cerebellar white matter is more salient.

### 3.4. Effects of Parameters

**Figure 8:**
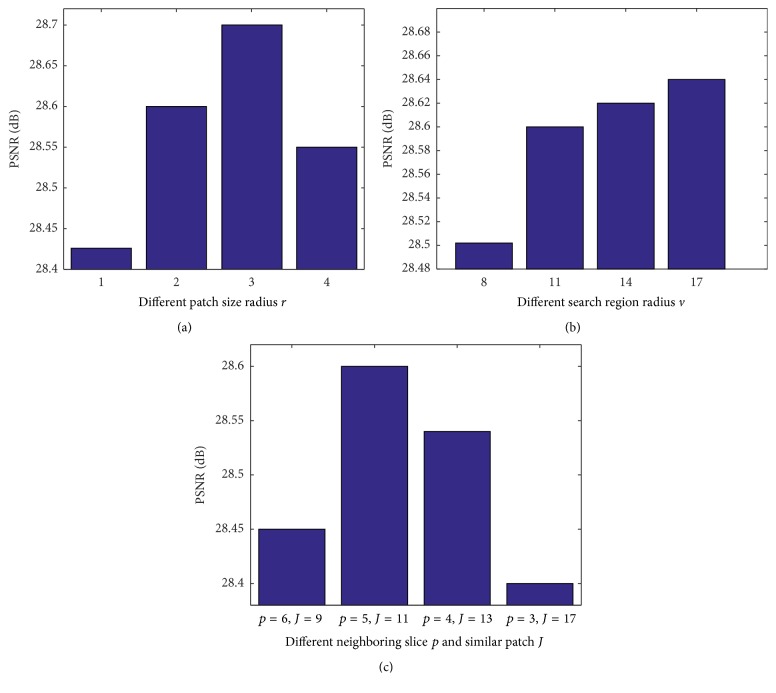
Performance comparison with different parameter settings. (a) Patch size radius, (b) search region radius, and (c) neighboring slice and number of similar patches in each slice. Note that, for each changing parameter, the other three parameters are fixed.

The proposed method has four tunable parameters: the radius (*v*) of the search region, the radius (*r*) of the 2D patch, the number (*p*) of neighboring slices, and the number (*J*) of the most similar 2D patches from each slice. Intuitively, large *v* helps to find more similar patches that can be used in derivative estimation. Large *r* tends to blur image details for edge regions, but too small *r* may cause annoying artifacts in smooth areas. Parameters *p* and *J* determine the number of weighted-square equations in (5), thereby influencing the accuracy of derivative estimation. To investigate how these four parameters affect upsampling performance, we take a 2 × 2 × 2 mm^3^ LR volume (with no noise) generated by BrainWeb as example to probe the selection.  Figure 8 shows the changing results of PSNR and SSIM varying with different parameter settings.

Regarding patch size radius, using patch size of 7 × 7 yields the highest PSNR value. Nevertheless, this patch size greatly increases computational load, the running time of which is ten times more than using a 3 × 3 patch. As for the search region size, we see from Figure 8(b) that a larger search region leads to a higher PSNR value, which makes sense since patches with higher similarity are more likely to be found within a large search region. Regarding leveraged neighboring slice and the number of patches in each slice, two interesting phenomena are observed. (1) Using a greater number of neighboring slices reduces PSNR value. This is because the image slice that is not near the target may not have the same geometric layout, and thereby the “similar” patches found in this slice may introduce large errors in the derivative estimation. (2) Finding a greater number of similar patches in one slice reduces PSNR value as well. This is because if more patches are exploited inside one image, some of them may not resemble the target. Therefore, to balance reconstruction quality and time efficiency, from the above empirical study, it is proven that *r* = 2, *v* = 11, *p* = 5, and *J* = 11 may be proper for MR images in our upsampling model.

## 4. Conclusion

A new high-order regression-based framework was proposed in this paper for a high quality MR image upsampling process. Prompted by several recently popular interpolation-based image upsampling methods in MR imaging [2, 5–11], the proposed method first concludes that these methods all belonged to a zeroth-order regression framework, which would jeopardize the recovery of image's fine details such as the laminar shape of brain structures. Regarding this, the proposed method extends the traditional zeroth-order framework into the second order by applying a Taylor expansion on each MR image. Then, in order to obtain a robust second-order regression function estimation, not only self-similarity property in a single MR image but also intrasimilarity property in adjacent MR slices is employed. Furthermore, unlike traditional interpolation-based methods which estimate the HR volumes voxel-by-voxel, the proposed method was performed in patches, which enforces region conformity in the reconstructed results.

The proposed method has been demonstrated, using synthetic and real data, to outperform both NLM and GPR methods, the state-of-the-art image upsampling methods, visually and quantitatively. Experiments on BrainWeb images show that, even under an ill-posed scenario (e.g., for 3x upsampling) or a noisy image (e.g., with the noise level at 9% of the maximum intensity), our method was able to reconstruct vivid details without introducing obvious artifacts. Moreover, the superior experimental results on the NAMIC, HCP, and clinical data implied that the proposed method can be applied to real applications.

While in this investigation we are only focused on noise-free brain MR images, the proposed method relies on a predenoising step to deal with noisy MR images. However, from the experimental results for the GPR method and the NLM method shown in Figure 4, we can see that a simultaneous interpolation and denoising eventually outperform an asynchronous framework. This performance can be expected because the extra denoising step would inevitably weaken image's details. To this end, we would like to investigate a combination of interpolation and denoising in our future work, which, we believe, would further improve the image's upsampling result.

## Figures and Tables

**Algorithm 1:**
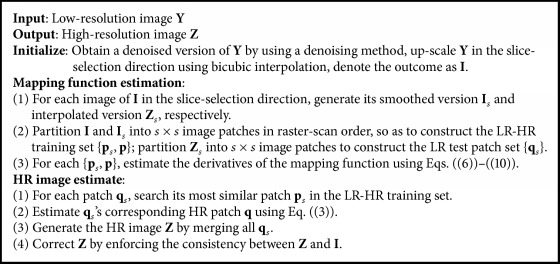
Regression-based MR image upsampling.

**Table 1:** PSNR, SSIM, and MI values for different methods on different slice thickness (PSNR/SSIM/MI).

	1 × 1 × 2 mm^3^	1 × 1 × 3 mm^3^	1 × 1 × 5 mm^3^	1 × 1 × 6 mm^3^
NN	25.62/0.81/1.59	24.09/0.78/1.44	21.78/0.72/1.21	21.68/0.69/1.11
GPR	25.80/0.83/1.61	24.12/0.82/1.45	21.89/0.74/1.22	21.78/0.72/1.13
NLM	26.43/**0.87/**1.61	24.74/0.82/1.47	22.56/0.75/**1.29**	22.30/0.72/1.16
Ours	**26.56**/**0.87/1.62**	**24.96**/**0.83/1.48**	**23.62**/**0.76/1.29**	**22.90**/**0.73/1.18**
